# Morphostructural MRI Abnormalities Related to Neuropsychiatric Disorders Associated to Multiple Sclerosis

**DOI:** 10.1155/2013/102454

**Published:** 2013-04-16

**Authors:** Simona Bonavita, Gioacchino Tedeschi, Antonio Gallo

**Affiliations:** ^1^Second University of Naples, II Clinic of Neurology, Piazza Miraglia 2, 80138 Naples, Italy; ^2^Neurological Institute for Diagnosis and Care “Hermitage Capodimonte”, Naples, Italy

## Abstract

Multiple Sclerosis associated neuropsychiatric disorders include major depression (MD), obsessive-compulsive disorder (OCD), bipolar affective disorder, euphoria, pseudobulbar affect, psychosis, and personality change. Magnetic Resonance Imaging (MRI) studies focused mainly on identifying morphostructural correlates of MD; only a few anecdotal cases on OCD associated to MS (OCD-MS), euphoria, pseudobulbar affect, psychosis, personality change, and one research article on MRI abnormalities in OCD-MS have been published. Therefore, in the present review we will report mainly on neuroimaging abnormalities found in MS patients with MD and OCD. All together, the studies on MD associated to MS suggest that, in this disease, depression is linked to a damage involving mainly frontotemporal regions either with discrete lesions (with those visible in T1 weighted images playing a more significant role) or subtle normal appearing white matter abnormalities. Hippocampal atrophy, as well, seems to be involved in MS related depression. It is conceivable that grey matter pathology (i.e., global and regional atrophy, cortical lesions), which occurs early in the course of disease, may involve several areas including the dorsolateral prefrontal cortex, the orbitofrontal cortex, and the anterior cingulate cortex whose disruption is currently thought to explain late-life depression. Further MRI studies are necessary to better elucidate OCD pathogenesis in MS.

## 1. Introduction

Neuropsychiatric disorders associated to Multiple Sclerosis (MS) may be divided into two categories: mood disorders (including behavioural disturbances) [1] and cognitive impairment. The high preponderance of psychiatric symptoms in patients with MS has led to the suggestion that this disease should be routinely included in the differential diagnosis of patients being seen for psychiatric complaints [2–4]. Diaz-Olavarrieta et al. [5], by administering the Neuropsychiatric Inventory [6] to 44 patients with MS who were not under steroid treatment or were not under relapse, found that 95% of the patients were experiencing neuropsychiatric symptoms; in particular, dysphoria or depressive symptoms were most common (79%), followed by agitation (40%), anxiety (37%), irritability (35%), apathy (20%), euphoria (13%), disinhibition (13%), hallucinations (10%), and delusions (7%).

In this review we will focus on MS associated mood and behavioural disorders and, in particular, on their neuroimaging aspects.

MS associated neuropsychiatric disorders include major depression (MD), obsessive-compulsive disorder (OCD-MS), bipolar affective disorder, euphoria, pseudobulbar affect, psychosis, and personality change. 

So far, neuroimaging studies on MS focused mainly on identifying morphostructural changes typical of the disease and on recognizing predictors of disability and/or cognitive impairment. A more limited number of articles report on neuroimaging of psychiatric aspect of MS, with the majority of them concerning the morphostructural correlates of MD associated to MS. To date, MRI studies include only a few anecdotal cases on OCD-MS, euphoria, pseudobulbar affect, psychosis, personality change, and one research article on OCD in MS patients. 

In the present review we will report mainly on neuroimaging abnormalities found in MS patients with MD and OCD.

## 2. Depressive Disorders 

The most common psychiatric syndrome in MS, and also in general population, is MD, which is defined by the fourth edition of the Diagnostic and Statistical Manual of the American Psychiatric Association as follows.

Five or more of the following symptoms over a minimum two week period:

- depressed mood for most of the day,
- markedly diminished interest or pleasure in all activities,
- significant weight loss, or weight gain (5% of body weight in a month),
- insomnia or hypersomnia nearly every day,
- psychomotor retardation or agitation (observable by others),
- fatigue or loss of energy nearly every day,
- feelings of worthlessness, excessive inappropriate guilt,
- diminished ability to think or concentrate,
- recurrent thoughts of death.

A point prevalence of 15% to 30% and a lifetime prevalence of 40%–60% of MD have been reported in MS patients; this rate of depression is 3 to 10 times that of the general population [7]. The factors responsible for mood disturbances in MS are not well established: a psychological reaction to a progressively disabling and unpredictable disease may be a relevant contributor but reactive mechanisms alone are probably not sufficient to account for the high prevalence and wide spectrum of depression. Currently the physiopathology of MS-related depression is thought to be multifactorial and neuroinflammatory, neuroendocrine and neurotrophic mechanisms have been advocated [8]. The association between affective illness and MS is well described and to clarify whether mood dysregulation follows familial patterns of transmission similar to those found in patients with primary affective illness, Joffe et al. [9] assessed the prevalence of psychiatric diagnoses in the relatives of patients with MS, using the family history approach to diagnosis [10]. They found that the prevalence of psychiatric disorders, particularly affective illness, in the first degree relatives of patients with MS is consistent with that reported for the general population suggesting that affective disorders in this disease may be an intrinsic part of the neurological disorder rather than an independently acquired psychiatric disorder. Driven by these considerations there have been many attempts to identify the anatomical correlates explaining MD in MS patients. 

Neuroimaging studies in patients with MS have revealed associations between brain abnormalities and depression; computed assisted tomography studies reported that patients with brain lesions were more depressed than patients with only spinal cord involvement [11]. Other studies investigated the correlation between lesions location and occurrence of depression. Pujol et al. [12] studied 45 patients by MRI and tested the presence of depression by the Beck Depression Inventory (BDI). Axial spin-echo sequences were used to quantify lesions observed in three major anatomic divisions (basal, medial, and lateral) of the frontotemporal white matter (WM) in each hemisphere. These regions were chosen because they involve different cerebral connections but include the main frontotemporal associations bundles (considered to be involved in MD pathogenesis): the uncinate fasciculus (basal), the cingulum (medial), and the arcuate fasciculus (lateral). The authors found that the BDI scores significantly correlated with lesion load (LL) of the left arcuate fasciculus region (which contains neocortical-neocortical connections) of the left “verbal” hemisphere; in particular lesions in this area accounted for 17% of depression score variance. No significant correlation was found between lesions in the regions of the right hemisphere (dominant in regulating emotional behaviour) and depression, suggesting that misregulation of emotional behaviour and depressed mood are two different phenomena produced by different mechanisms. 

Subsequently, Feinstein et al. [13] evaluated MS subjects with and without depression by MRI; automatic tissue segmentation (grey matter (GM), WM, and cerebrospinal fluid (CSF)) and regional brain masking were applied to MRI data; regional and total LL were also calculated. They found that depressed MS patients had more hyperintense lesions in the left inferior medial frontal regions and greater atrophy of the left anterior temporal regions; they postulated that a combination of inflammation, demyelination, and atrophy within medial inferior frontal areas would disconnect neural connectivity with an associated perturbation in several neurotransmitters and modulators known to be involved in frontal subcortical circuits, with the monoaminergic ones being the most intimately tied to disorders of mood. Alterations of afferent and efferent connections from frontal subcortical areas to temporal lobe limbic areas may play a significant role in mood regulation as well.

A qualitative “visual” assessment of T1, T2 LL, and brain atrophy performed by Bakshi et al. [14] showed that the only MRI abnormalities that could significantly predict the presence of depression, before and after adjusting for EDSS, were T1 LL in superior frontal, superior parietal, and temporal regions, while the severity of depression was predicted by T1 lesions in superior frontal, superior parietal and temporal regions, enlargement of lateral and third ventricular, and frontal atrophy. The authors speculated that WM lesions in MS patients, especially those in frontal and parietal areas, lead to depression by disconnection of brain cortical areas that regulate the mood; in particular, frontal WM disease in MS could have effects on serotoninergic pathways in frontal-limbic circuits. Moreover, since T1 hypointensities appear to represent more specific chronic destructive irreversible tissue changes such as hypocellularity, complete demyelination, and axonal loss, the association between depression and T1 lesions, indicates that chronic irreversible damage to critical pathways is more likely to cause mood dysfunction. On the other hand, these pathways can function sufficiently well in the presence of less severe insults (edema, partial demyelination, and inflammation) as reflected by T2 LL. Furthermore, brain atrophy measures associated to depression suggest that patients with depression have more severe neuropathologic subsets of MS with a tendency towards more tissue destruction and thus may be more susceptible to dysfunction of mood regulating pathways.

An MRI study of 95 consecutive MS patients [15], in which 19% of the patients met the criteria for MD, reported that presence of MD and severity of depression were correlated with right frontal LL and with right temporal brain atrophy; furthermore, T1 lesions in the superior parietal and superior frontal regions predicted depression in MS patients. Of note, these 95 MS patients were also tested for anxiety by the Hamilton Anxiety Rating Scale (HARS) and the HARS scores did not correlate significantly with any of the MRI measures of regional and total LL and brain atrophy. The same authors performed a two-year follow-up study [16] to determine whether changes in total or regional LL and brain atrophy in specific regions of the brain may contribute to the development of depression in patients with MS. Brain atrophy evolution was significantly more conspicuous in the left frontal lobe of depressed patients as compared to nondepressed patients. The correlation analysis, considering the 2-year changes of MRI quantitative measures of regional and total LL and brain atrophy and the 2-year changes of the Hamilton Depression Rating Scale score, showed a significant correlation between the latter and right temporal brain atrophy; moreover, changes in right temporal brain atrophy were significantly and independently related to the severity of depressive symptoms. The authors suggested that in MS the temporal lobes involvement (and the subsequent damage in the main projection areas to the limbic system) may play a role in the aetiology of depressive mood disorders. 

More recently, a diffusion tensor imaging (DTI) study [17] investigated normal appearing brain tissue in the attempt of shedding further light on the pathogenesis of depression in patients with MS. T1 and PD/T2 LL were evaluated; tissue segmentation (normal appearing white (NAWM) and grey matter (NAGM), CSF), regional atrophy, and DTI analysis were performed as well. Depressed patients had a smaller NAWM volume in the left superior frontal region and a greater T1 LL in the right medial inferior frontal region. Significantly higher mean diffusivity was found in the depressed group in the NAGM of the left anterior temporal lobe region. Reduced fractional anisotropy (FA) was present in the NAWM of the left anterior temporal lobe in the depressed group. In addition, higher mean diffusivity was found in hyperintense lesions in the right inferior frontal region of the depressed group. These findings provide important data on structural brain changes beyond that captured by lesion and atrophy measurements and suggest that when inflammation and demyelination disrupt cellular organization and the linearity of fibres pathways in specific brain regions, even in the absence of discernable lesions, clinical depression may result. Therefore, it was concluded that more destructive aspects of brain change, that is, the black holes of T1-weighted images and reduction in NAWM volume are strictly related to mood disorders in MS patients.

Since a smaller volume of the hippocampus was found in psychiatric patients with MD, Gold et al. [18] investigated patients with MS and MD to explore whether subregional volumes of the hippocampus may be associated with the high frequency of depressive symptoms in MS.

In this sophisticated study the authors performed hippocampal structural segmentation identifying four regions: cornu ammonis 1 (CA1), CA2-CA3 and dentate gyrus (CA23DG), subiculum, and entorhinal cortex; global atrophy and lesion quantification were evaluated as well. In MS patients with depressive symptoms smaller CA23DG volumes were found (as a distinctive pattern of regional hippocampal atrophy); the correlation analysis revealed an inverse correlation between BDI scores and CA23DG volumes. The authors concluded that some forms of depression in MS may be caused by similar mechanisms hypothesized for MD in psychiatric patients. Moreover, since recently it has been hypothesized a neuroendocrine-limbic aetiology of depression and at the same time there is accumulating evidence that the hypothalamic-pituitary-adrenal axis (HPA) activity is increased in MS patients [19], Gold et al. [18] tested whether specific subregional volumes may be linked to alterations in diurnal cortisol secretion. They found that MS depressed patients, as compared to not depressed patients, had cortisol hypersecretion (elevated evening levels and unchanged cortisol levels in the morning) indicating that diurnal cortisol flattening, due to elevated evening cortisol (i.e., failure to decrease cortisol responses throughout the day), was associated with depression in MS and not with MS. Furthermore, there was an inverse correlation between CA23DG volumes, BDI, scores and cortisol levels. The authors suggested that even subtle hyperactivity of the HPA axis may produce smaller volumes in the CA23DG region and thereby lead to depressive symptoms in MS. The same authors, by using surface mapping techniques, performed volumetric and shape analyses of the hippocampus to characterize neuroanatomical correlates of depression in MS [20]. Two groups of MS patients, respectively with low and high depression scores were studied. Right hippocampal volumes were smaller in the high depression versus the low depression group, but there were no significant differences in left hippocampal volumes. Since in this study only female patients were included, and one recent study on familial depression with a sample including only female patients showed reduced volumes of the right but not the left hippocampus [21], the authors interpreted their finding as an indication for sex-specific associations between lateralized hippocampal atrophy and depression.

It should be noticed that all the above-mentioned studies are not free from limitations: the majority of them did not take into account possible confounders like fatigue, cognitive impairment, and number of previous steroids cycles; only some of them included a healthy control group, nonetheless an appropriate protocol design including also psychiatric depressed patients was never done. Furthermore, in these studies depression was evaluated by using depression scales mainly validated for psychiatric patients and not for MS patients in whom physical symptoms may be possible confounders for depression assessment.

All together these studies suggest that depression in MS is linked to a damage involving mainly frontotemporal regions either with discrete lesions (with those visible in T1 weighted images playing a more significant role) or subtle NAWM abnormalities. Hippocampal atrophy, as well, seems to be involved in MS related depression. 

The frontal lobe, and in particular the prefrontal cortex, is involved in information processing. The prefrontal cortex role includes planning, organization, inhibition, empathy, and motivation that are dependent on three distinct cortical networks: (i) the dorsolateral prefrontal cortex, (ii) the orbitofrontal cortex, and (iii) the anterior cingulate cortex [22, 23]. Axons project from these cortical areas, through the WM, to subcortical structures, and MS plaques, involving mainly WM (where the fibre tracts travel extremely close), may disrupt these circuits impacting deeply on their function. The dorsolateral prefrontal cortex is involved in executive functioning (i.e., planning, organization, and attention) and its damage is responsible for perseveration, difficulty in shifting and screening out environmental distractions, impairments in constructional skills, and sequential motor tasks. The orbitofrontal cortex controls socially appropriate behaviour and empathy, thus MS lesions may result in impulsivity, lability, personality changes, and lack of humanistic sensitivity. The anterior cingulate cortex is thought to have at least two further subdivisions: the affect and the cognition ones [24]. The affect portion has connections with the limbic and paralimbic regions, including the orbitofrontal cortex. The cognition subdivision connects with the parietal cortex, the spinal cord, and the dorsolateral prefrontal cortex. These connections highlight the linkage and interdependence of the frontal circuits. Disruption of the aforementioned brain circuitry is thought to explain late-life depression. Indeed, according to this hypothesis, Taylor et al. [25] by using statistical parametric mapping identified frontal WM lesions in elderly depressed patients and found an association between lesions of WM tracts extending from inferior frontal regions toward the basal ganglia and depression. Alexopoulos and coauthors [26, 27] have defined the “depression-executive dysfunction syndrome” in the elderly, which is thought to be a distinct depressive disorder marked by executive dysfunction as a result of lesions in the basal ganglia and left frontal regions. 

Thus, it can be hypothesized that MS patients, generally in young adult age, by having WM plaques disrupting these cortical networks, are at higher risk of developing depression than healthy peers. 

Furthermore, new MRI acquisition and image analysis techniques, currently being used for research purposes, such as DTI, tract-based spatial statistics (TBSS), magnetization transfer imaging, Double Inversion Recovery sequences, voxel based (VBM) and surface based morphometry, lesion probability maps, SIENAX, and resting state functional MRI, by allowing a more extensive study of both WM and GM, outside visible MS plaques, will probably help in better understanding the specific role of GM and WM in the pathogenesis of affective disorders associated to MS. 

**Figure 1:**
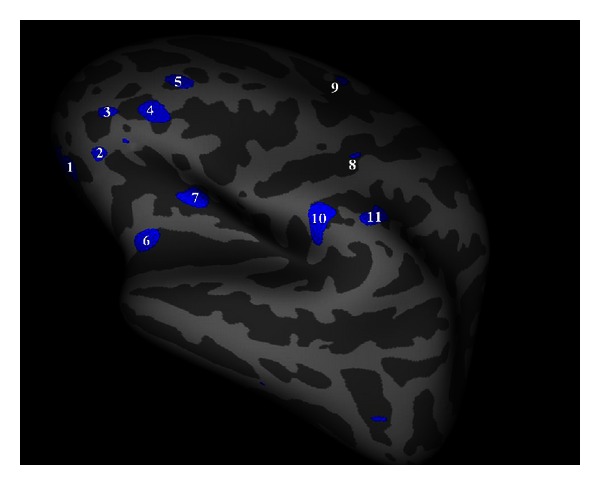
Left hemisphere, lateral surface. Cortical thickness group analysis between patients with MS related depression (MS-dep) and not depressed MS patients. Areas showing a significant cortical thinning in patients with MS-dep (*P* < 0.01) are coloured in blue. Area 1 is localized in orbito-frontal cortex; areas 2, 3, 4, 5 are localized in prefrontal cortex; areas 10 and 11 are localized in parietal cortex.

In particular, surface based morphometry may be suitable for mapping regional cortical thickness in the brain networks presumably involved in MS related depression (see Figure 1, personal data).

## 3. Obsessive-Compulsive Disorder (OCD)

The lifetime prevalence of OCD in MS is 8.6% compared with 2.5% in the general population [28]. In primary OCD, neuroimaging studies have revealed structural and/or functional abnormalities in specific brain structures, particularly in the fronto-striato-thalamic circuitry [29–31] which points to an organic aetiology of this psychiatric disease. 

In an MS patient, OCD developed after being diagnosed with MS concurrently with the appearance of a right parietal WM plaque [32]; this single case raised the possibility that parietal lobe WM microstructure abnormalities play a role in mediating obsessions and compulsions through disruptions of the functional connectivity between cortical-cortical and/or cortical-subcortical brain regions implicated in the pathophysiology of OCD.

Recently, Tinelli et al. [33] evaluated by MRI the relationship between GM and WM tissue damage and OCD in patients with MS. The authors studied 16 MS patients with and 15 without OCD. Image analysis included LL, VBM, FA calculation, and TBSS. The only significant differences were found in the VBM analysis that revealed a set of clusters of reduced GM volume in patients with OCD in the right inferior frontal gyrus, inferior temporal gyrus, and middle temporal gyrus. The absence of any significant difference in TBSS analysis in the two groups seems to be in contrast with the results of recent DTI studies in primary OCD, which have reported abnormalities in the corpus callosum and subcortical frontal WM associated with either increased [34] or decreased [35] structural connectivity.

Probably, differences in patients' characteristics and/or in methods of MRI acquisition and data analysis could account for this discrepancy thus leaving space to further studies to better elucidate OCD pathogenesis in MS.

In conclusion, despite the great advancements achieved so far, we anticipate that the application of modern advanced MRI technologies and better definition of patients' features will prompt us to get significantly closer to a more intimate knowledge of the physiopathology and functional basis of neuropsychiatric disorders in MS.
